# Surface determination through atomically resolved secondary-electron imaging

**DOI:** 10.1038/ncomms8358

**Published:** 2015-06-17

**Authors:** J. Ciston, H. G. Brown, A. J. D'Alfonso, P. Koirala, C. Ophus, Y. Lin, Y. Suzuki, H. Inada, Y. Zhu, L. J. Allen, L. D. Marks

**Affiliations:** 1 National Center for Electron Microscopy, The Molecular Foundry, Lawrence Berkeley National Laboratory, Berkeley, California 94720, USA; 2 School of Physics, University of Melbourne, Parkville, Victoria 3010, Australia; 3 Department of Materials Science and Engineering, Northwestern University, Evanston, Illinois 60208, USA; 4 Application Development Department, Hitachi High Technologies Corp., Ibaraki 312-8504, Japan; 5 Advanced Microscope Design Department, Hitachi High Technologies Corp., Ibaraki 312-8504, Japan; 6 Condensed Matter Physics and Materials Science, Brookhaven National Laboratory, Upton, New York 11973, USA

## Abstract

Unique determination of the atomic structure of technologically relevant surfaces is often limited by both a need for homogeneous crystals and ambiguity of registration between the surface and bulk. Atomically resolved secondary-electron imaging is extremely sensitive to this registration and is compatible with faceted nanomaterials, but has not been previously utilized for surface structure determination. Here we report a detailed experimental atomic-resolution secondary-electron microscopy analysis of the c(6 × 2) reconstruction on strontium titanate (001) coupled with careful simulation of secondary-electron images, density functional theory calculations and surface monolayer-sensitive aberration-corrected plan-view high-resolution transmission electron microscopy. Our work reveals several unexpected findings, including an amended registry of the surface on the bulk and strontium atoms with unusual seven-fold coordination within a typically high surface coverage of square pyramidal TiO_5_ units. Dielectric screening is found to play a critical role in attenuating secondary-electron generation processes from valence orbitals.

## Abstract (web summary)

Technical difficulties have so far limited the application of high-resolution secondary-electron microscopy in imaging surface structures. Here, the authors report a successful determination of surface reconstruction of strontium titanate, using the secondary-electron microscopy along with other techniques.

## Graphical abstract

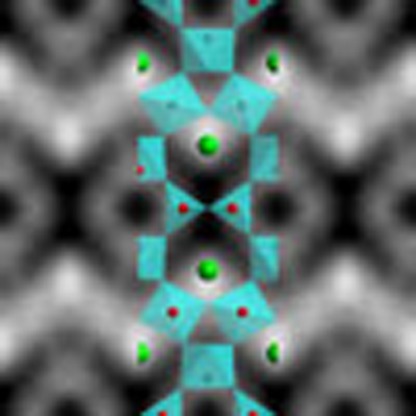

A promising new approach to the determination of surface structures is high-resolution secondary-electron microscopy (HRSEM), where an aberration-corrected, Ångstrom scale electron probe is rastered across the sample and the low-energy (0–20 eV) electron signal emitted from the surfaces is collected^1^. Initial demonstrations of the technique exhibited enhanced surface sensitivity over other atomic-resolution transmission electron microscopy techniques, but to date this has never been exploited to image surface structures that differ from the underlying bulk.

To draw robust conclusions about surface structure from HRSEM images, it is crucial to compare the experimental data with a rigorous model for HRSEM image generation. The first attempt to model the imaging mode assumed that secondary electrons were due to some form of inelastic scattering with a Z^0.53^ dependence^2^. That method successfully reproduced the relative atomic contrast of experimental HRSEM data suggestive of bulk-like SrO and TiO_2_ terminations on SrTiO_3_ (ref. 3). A more rigorous model, assuming that the secondary electrons are ejected in ionization events, and including the angle-dependent probability of electron ejection was subsequently developed by Brown *et al*^4^. This formalism did not contain adjustable parameters and produced a much better qualitative match to both the relative atomic column intensities and delocalized contrast in experimental HRSEM data from YBa_2_Cu_3_O_7−*x*_ dominated by bulk secondary-electron emission. However, the model of Brown *et al.* was not fully tested to determine the surface selectivity of HRSEM, and only considered the contribution of significant core level ionizations to the signal. As we will demonstrate, additional physics must be incorporated in the secondary-electron generation model to properly extend the technique to represent the subtle perturbations to the bulk HRSEM signal generated by a well-ordered surface reconstruction. These extensions to the model describe fundamental physics and do not rely on any adjustable free parameters.

An important problem in understanding surface structures is determining how the outermost layers are registered with respect to the underlying bulk, which is often ambiguous. The combination of HRSEM and annular dark field (ADF) STEM is uniquely well suited to solve the registration problem, as it allows for unambiguous simultaneous measurement of both the atomically resolved bulk (ADF) and surface (HRSEM) crystal structure without reliance on subtle phase interference effects. Although diffraction-based phase inversion techniques can in principle determine registration, it is not a trivial undertaking and requires an extensive search over a large solution space to find the phase combination that is most consistent with experimental data. Even if a successful inversion is performed the interpretation of the results is not always straightforward^5^. Alternative approaches such as proximal scanning probe methods are invaluable for determining the outermost surface structure but they are rarely sensitive to the bulk registry for a continuous reconstruction. Plan-view high-resolution transmission electron microscopy (HRTEM) has been demonstrated for known surface structures^6^,^7^,^8^,^9^,^10^,^11^, but the number of successful applications to unknown surface structures is very small^12^,^13^. Cross-sectional HRTEM techniques can also provide important information about bulk–surface registration, but cannot account for the possibility of multiple surface domains in the projection of the cross-section.

In this article, we report a detailed analysis of the c(6 × 2) reconstruction on the surface of SrTiO_3_ (001) using HRSEM images coupled with a range of other techniques. The HRSEM is uniquely sensitive to the registry of the reconstruction on the underlying bulk, and turns out to be very sensitive to dielectric screening not included previously and also disorder at the surface. The structure we find for the surface is consistent with all the prior experimental data as well as our new HRSEM and HRTEM data. The HRSEM image simulations without adjustable parameters have a Pearson correlation score of 0.95 compared with the experimental data, comparable to the level of quantitative confidence of current simulations of ADF and HRTEM images.

## Results

### Brief overview of topics covered

The expectation when this work started was that we would be comparing the HRSEM contrast to that of a reconstruction of the SrTiO_3_ surface for which a structure had previously been reported. However, it was very quickly apparent, due to the unambiguous ability of HRSEM to handle issues related to registry of the surface on the bulk, that this earlier model was incorrect. This was despite it matching quite well to available scanning tunnelling microscopy (STM) images and surface X-ray diffraction data. In the following sections, we will progressively analyse the results dealing first with the registry and modifications to the prior model that yielded a structure consistent with all the available experimental data before turning to discuss the fine details of the HRSEM images and quantitative simulation thereof.

**Figure 1:**
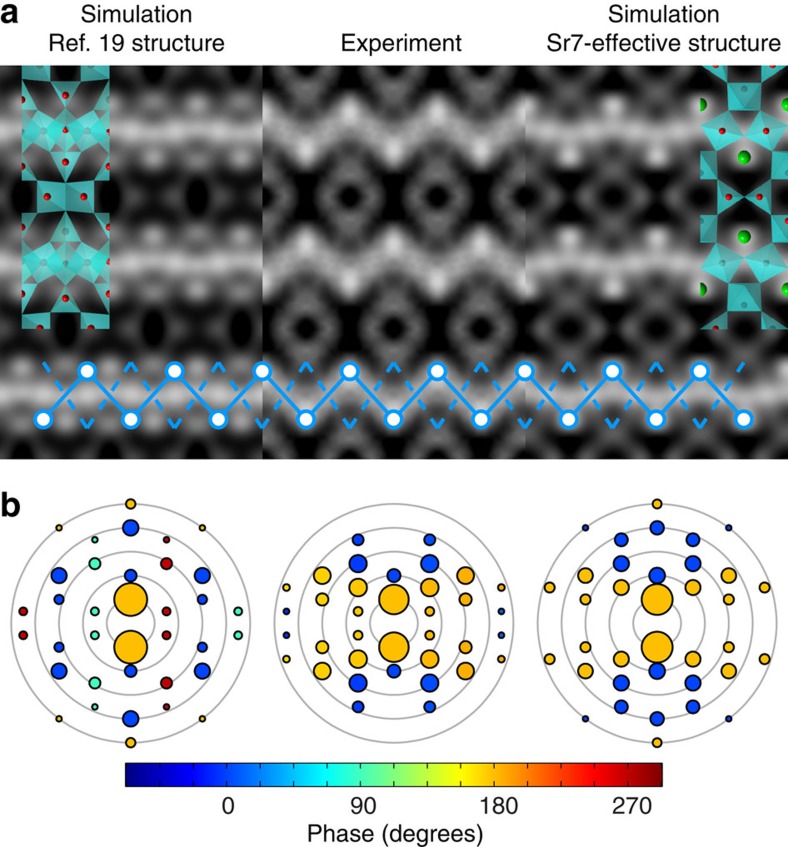
Direct determination of bulk–surface registration through HRSEM images. (**a**) Comparison of the (centre) bulk-subtracted experimental HRSEM image to simulated bulk-subtracted HRSEM images from the (left) rumpled vacancy surface reconstruction as reported in ref. 19 and (right) new Sr7-effective c(6 × 2) reconstruction reported in this work. Zigzag lines are to guide the eye and clearly show the misregistration of the ref. 19 structure. All bulk-subtracted images are referenced to the same origin of the underlying bulk unit cell. Each image panel measures 2.34 × 3.51 nm. (**b**) Fast Fourier transforms of HRSEM images from (left) simulation of the ref. 19 reconstruction (centre) experimental and (right) simulation of new c(6 × 2) Sr7-effective reconstructions. Circle area is proportional to the FFT amplitude, and colours are indicative of phase. Note the red/green phase asymmetry in the FFT of the ref. 19 structure. Details of the bulk subtraction procedure are discussed in the Methods section.

Single crystal samples of 0.7% wt Nb-doped SrTiO_3_ (001) were thinned and annealed to produce the c(6 × 2) surface^14^ with atomically flat terraces as confirmed by weak-beam dark-field imaging and transmission electron diffraction (TED) as described further in the Methods section (Supplementary Fig. 1). These were then examined using an aberration-corrected Hitachi HD2700C^15^ at the Hitachi High Technologies Science Laboratory to obtain secondary-electron images with simultaneously acquired ADF images to elucidate the registration to the bulk. The images were processed as discussed in the Methods section and the bulk-subtracted images and corresponding simulations for two candidate structures are shown in Fig. 1a. An identical surface reconstruction was found on all four independent sample terraces in the HRSEM field of view (Supplementary Fig. 2). Experimental measurements with an *in situ* biasing holder confirmed that secondary electrons with energy <20 eV contribute >90% of the signal in the SE detector (Supplementary Fig. 3). Monochromated aberration-corrected HRTEM imaging using the TEAM 0.5 instrument^16^ at the National Center for Electron Microscopy facility of the Molecular Foundry at Lawrence Berkeley National Laboratory was also performed. The HRTEM images were processed using the methodology previously used for the silicon 7 × 7 (111) reconstruction^11^ to separate the top and bottom surfaces; this has now been automated within the EDM code^17^. As a cross-check of the separation procedure, HRTEM images were simulated using the MacTempas code^18^. More details can be found in the Methods section.

### Direct measurement of bulk/surface registration

A possible c(6 × 2) surface reconstruction consistent with the canonical double layer TiO_2_ termination on SrTiO_3_ (100) has previously been published in ref. 19. In that work, a complex structural model incorporating fractional contributions from four structurally related reconstructions was found to be consistent with three-dimensional (3D) surface X-ray diffraction data, TED data and STM images. While the simulated HRSEM images using these prior structures (left side of Fig. 1a) showed a superficial agreement to the bulk-subtracted experimental data (Fig. 1a, centre), closer analysis indicated that the registry of the surface atoms was shifted by (½, 0) with respect to the 1 × 1 SrTiO_3_ bulk unit cell by inspection of both the real space images and the phases of their respective Fast Fourier Transforms (FFTs) as shown in Fig. 1. This registration shift is incontrovertible due to the internal structural references of both the total HRSEM signal and the simultaneously recorded ADF signal, which both clearly distinguish Sr and Ti bulk atomic sites, proving that the previously published structure is unambiguously incorrect. The ability to rule out entire classes of surface structures by inspection of the bulk/surface registration is one of the primary advantages of the HRSEM technique.

To reproduce the shifted registry while maintaining consistency with HRSEM, surface X-ray diffraction, TED and STM data, DFT calculations were performed for about 25 variants. We found an improved structure for the c(6 × 2) reconstruction, hereafter referred to as Sr7, consisting of a triple TiO_2_ overlayer with two Sr atoms with seven-fold coordination substitutionally exchanged for Ti in the uppermost surface layer, shown in Supplementary Fig. 4. (Atomic positions are included in Supplementary Note 1 as a Crystallographic Information Format (CIF) file.) The third TiO_2_ layer close to bulk positions yields the required registry shift while retaining almost the same external surface that STM sees, and this additional layer in bulk-like positions has little effect on the surface X-ray diffraction and TED intensities. The Sr7 structure is stable in DFT calculations with reasonable bond distances and includes a high surface coverage of TiO_5_[] octahedrally coordinated titanium with a vacant oxygen site ‘[]' similar to other known reconstructions on the (001) surface^20^,^21^,^22^.

The right side of Fig. 1a shows an HRSEM simulation for the Sr7 structure after the effects of disorder have been accounted for, referred to as Sr7-effective. This structure maintains the correct registry to the bulk crystal and is in good quantitative agreement with the experimental HRSEM data, and will be discussed later in detail. Note that the FFT amplitudes in Fig. 1b are very similar for both simulated structures, but the correct registration of the Sr7 structure is easily observed by inspection of the FFT phases, which are not directly accessible through diffraction experiments.

### Confirmation of the Sr7 structure

**Figure 2:**
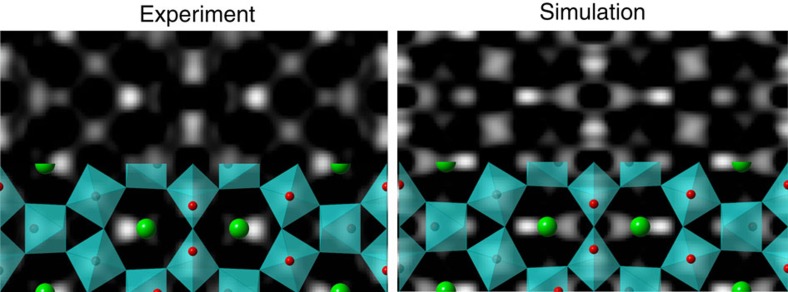
Comparison of bulk-subtracted experimental and simulated HRTEM images. Experimental (left) and simulated (right) HRTEM images of the SrTiO3 (001) c(6 × 2) Sr7 surface reconstruction on a 5.3-nm-thick sample at a defocus of 0.55 nm with bulk subtraction and corrected for the top/bottom surface registry shift with phases inverted 180 degrees for spatial frequencies where sin(*χ*)<0 in the phase contrast transfer function.

Since the Sr7 structure suggested by the HRSEM data has an unusual exposed strontium atom at the top of a surface that is more titanium-rich than any known reconstruction on strontium titanate, additional evidence from other experimental techniques is important. There is little uncertainty in the modelling of conventional high-resolution electron microscopy images, aside from the absolute contrast scale. Plan-view HRTEM surface imaging adds the complications of subtracting the bulk contribution, removing the effect of overlapping reconstructions on the top and bottom surfaces of the sample, and possible complexities due to dynamical scattering. While there are many approximations involved, the methodology used previously to separate the top and bottom overlap in the Si(111)-(7 × 7) system^11^ has been applied to the Sr7 structure and provides a best fit between HRTEM simulation and experiment at a thickness of 5.3 nm. The bulk subtraction for such a thin sample is not overwhelmed by dynamical effects at 80 kV and clearly distinguishes the relative contrast of Sr and Ti surface atoms in the double-backbone structural motif as shown in Fig. 2. Although the agreement is not perfect, it is reasonable considering the weak signal-to-noise level and clearly confirms the presence of a strongly scattering Sr atom unique to our Sr7 structure.

As a second check, the atomic coordinates of the Sr7 structure from DFT calculations, particularly for the positions of the strongly scattering metal atoms, were in good agreement with those refined against the 3D surface X-ray diffraction data utilized in ref. 19 with *R*_1_=0.2993 for the 694 structure factors larger than four times the measurement error, and 0.3379 for all 859 data (Supplementary Table 1). We also note that earlier Auger data^23^ suggested a high titanium excess for the c(6 × 2) reconstruction consistent with a three-layer model, although due to experimental complications an exact value for the coverage was not clear. STM simulations for the new Sr7 reconstruction are consistent with published data^23^, as shown in Supplementary Fig. 5.

**Figure 3:**
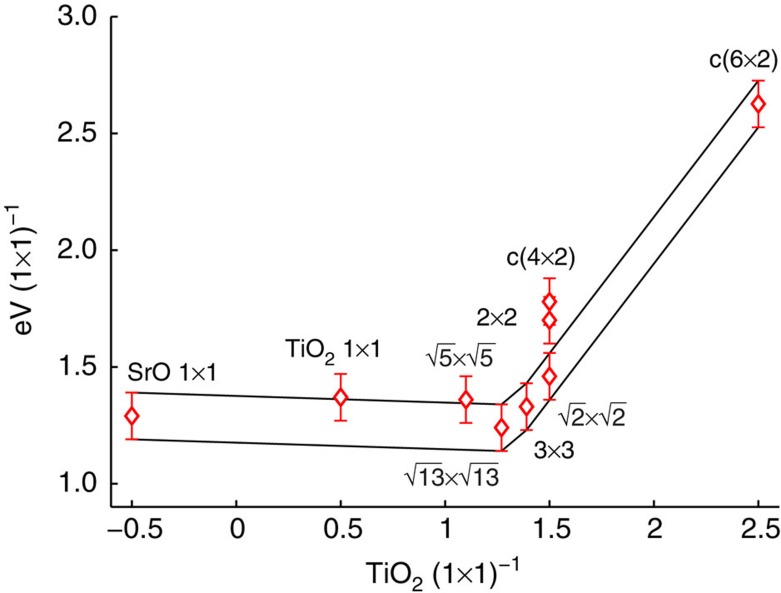
DFT convex hull of reconstructions on the (100) surface of SrTiO_3_. Units along the abscissa are excess TiO_2_ (1 × 1 cell)^−1^, and along the ordinate the energy in eV (1 × 1 unit cell)^−1^. Consistency checks between the different functionals indicated an error in the energies of ∼0.1 eV (1 × 1 cell)^−1^ (∼60 mJ m^−2^, 8 kJ mol^−1^) as described in the Supplementary Information. Therefore error bars of 0.1 eV (1 × 1 cell)^−1^ standard deviation are shown.

The last issue to resolve is whether the enthalpy of the surface structure is reasonable compared with other known surface reconstructions on SrTiO_3_ (100) with pure TiO_*x*_ terminations. The convex hull construction is shown in Fig. 3 (see also Supplementary Discussion), which includes all known reconstructions as well as the new c(6 × 2) structure. The enthalpy found for the newly proposed structure is consistent with other known reconstructions on SrTiO_3_ (001). The position as an end point on the convex hull poses somewhat relaxed constraints on the allowable energies of the c(6 × 2) structure. If the c(6 × 2) was substantially lower, the well documented c(4 × 2) would not be thermodynamically stable under any conditions. Furthermore, the c(6 × 2)  is sufficiently low to prevent separation into TiO_2_ islands at the surface, which is known to be possible at very high TiO_2_ surface coverage^24^.

### Optimization of HRSEM simulations

**Figure 4 f4:**
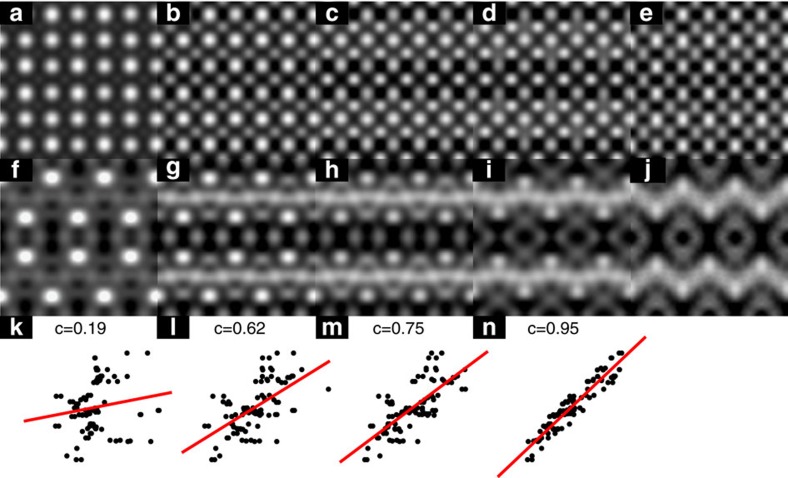
Comparison of HRSEM experimental and simulated images. (**a**–**c**) HRSEM simulations of the Sr7 structure including damping due to dielectric screening; (**a**) Using only core states (up to and including the 3*d* state in Sr, the 2*p* in Ti and 1*s* in oxygen). (**b**) Adding the 4*s* and 4*p* for Sr, the 3*s* and 3*p* for Ti and 2*s* for O. (**c**) Adding the contribution for a filled 2*p* orbital in O. (**d**) Shows the HRSEM simulation including all orbitals for the Sr7-effective structure. (**e**) Shows the experimental result with translational 6 × 2 unit cell averaging and c2 mm symmetry applied. (**f**–**j**) In this, we show the corresponding bulk-subtracted results for **a** through **e**. (**k**–**n**) In this, we show the Pearson product–moment correlation of image intensities corresponding to **f** through **j**. Details of the bulk subtraction procedure are discussed in the Methods section.

Simulation of the HRSEM images was performed using the formalism described in detail in ref. 4 with the important extension that all binding states were included, not just the deeper core states. We have also accounted for the damping of the ionization interaction due to dielectric screening. These extensions to the model presented in ref. 4 result in a more complete simulation of the HRSEM imaging process without adding any adjustable parameters. In the HRSEM imaging simulations, restricting consideration to just the deep core states as done earlier results in images that are dominated by the signal originating at the bulk strontium sites, see Fig. 4a,f. Supplementary Table 3 elucidates the relative contribution to the total HRSEM signal from each atomic orbital. In the case of this surface reconstruction, the valence O-2*p* and Sr-4*p* orbitals contribute >50% of the total HRSEM signal. Therefore, failing to include these orbitals for this case of a surface reconstruction would result in an inadequate description of the signal. It is worth remembering that while one often considers high-energy valence states to be delocalized, this only refers to the difference in charge density in the neutral atom and bonded cases; there is still a substantial local valence density around the atoms particularly when this is calculated using all-electron methods.

Appropriate treatment of the dielectric damping to the ionization potential was critical in achieving the match between theory and experiment for the HRSEM signal (compare Supplementary Fig. 6 to Fig. 4). The Fourier coefficients of the effective optical scattering potential for ionization (equation (3) in ref. 4) were modified to include the contribution of screening by other electrons for both the bulk and surface atoms—see Methods. Explicitly, the factor 1/*ɛ*_0_ in that equation, where *ɛ*_0_ is the permittivity of free space, was replaced by *F*(*ɛ*(Δ*E*)) × 1/*ɛ*_0_ with *ɛ*(Δ*E*) the complex dielectric function for an energy loss Δ*E*, where *F*(*ɛ*(Δ*E*)) is Re(1/*ɛ*(Δ*E*)) in the limit of pure bulk screening and Re(2/(1+*ɛ*(Δ*E*))) for pure surface screening. Both the bulk and surface limits were evaluated from the experiments in ref. 25 as described in the Methods section. This leads to an energy-dependent damping term with values given in Supplementary Table 2 (for those states where the damping is significantly different from unity) and a strong attenuation of the contribution from states near the Fermi energy.

When all the states are included, moderated by the dielectric screening, there is a reasonable agreement between the HRSEM simulations and experiment as indicated by Pearson product–moment correlation values shown in Fig. 4 and Supplementary Table 4. The agreement between the experiment and simulation validates the relatively straightforward treatment of secondary electrons escaping the sample, confirming that backscattering or complete inelastic absorption of liberated secondary electrons is a minor contribution to the HRSEM signal, which is dominated by the initial inelastic interaction of the swift electron probe. The HRSEM detector is relatively insensitive to the occurrence of dynamical forward scattering or inelastic losses of SEs leaving the sample due to integration over both energy and the full 2π steradian half space of electron trajectories above the sample surface for each local electron probe location. However, the quantitative agreement between simulation and experiment of the full Sr7 model in Fig. 4c,h is not as good as one normally expects for electron microscope images, which we will now show is due to disorder.

### Effects of local structural disorder

The HRSEM technique has clearly been shown to provide information about bulk/surface registration information and a degree of Z-contrast useful for the identification of the strongly scattering surface Sr atoms. However, due to signal-to-noise limitations with current first-generation detectors that are not yet fully optimized, a mean 6 × 2 unit cell was calculated from the experimental HRSEM data that represents a statistical average over the 60 × 60 nm^2^ field of view as described in the Methods section. STM images^23^ indicate that there is a substantial amount of disorder associated with adatoms, probably small TiO_2_ units as previously suggested^19^. Therefore, the HRSEM mean unit cell represents an ‘effective projected surface structure' and represents a convolution of the true periodic atomic positions and mean symmetrized local disorder, analogous to the information provided by the two-dimensional (2D) in-plane component of the surface X-ray diffraction data.

To determine atomic coordinates for this effective projected surface structure, the outermost Sr and Ti atoms of the Sr7 structure were refined against only in-plane surface X-ray diffraction data. The atomic positions shift slightly compared with the full 3D refinement (as expected) with a 0.7 Å displacement to the Sr atom and a 0.5 Å displacement for two of the four Ti atoms in the surface layer, see Supplementary Table 5. Full HRSEM simulations of the Sr7-effective structure are shown in Figs 1, 4d, 4i-4n, [Fig f4], [Fig f4], which clearly demonstrate an enhanced agreement with experimental HRSEM data and an improvement in the Pearson correlation score from 0.75 to 0.95. This demonstrates that HRSEM is very sensitive to small displacements in atomic positions.

## Discussion

The Sr7 structure we have found using this unique combination of methods, in particular, including the new tool of atomic-resolution secondary-electron imaging, is consistent with all the data that is available: surface X-ray diffraction, STM, Auger composition, HRTEM images, TED and DFT simulations. Within this arsenal of methods HRSEM has demonstrated that it can play a pivotal role in resolving issues concerning the registry of surface structures, where this is not easily achieved using other techniques. We have also shown that accounting for valence orbitals is important for HRSEM simulations, and that this requires incorporation of additional physics for local dielectric screening of the initial ionization events. Beyond this, one has to consider carefully exactly what the HRSEM images represent if there is disorder present, particularly with current first-generation detectors and averaging methods to overcome signal-to-noise issues.

Most of the current limitations of the method appear to be instrumental. For instance, there is room for increasing detection efficiency. The surface sensitivity of HRSEM could also be considerably improved by lowering the accelerating voltage to increase the scattering cross-section for ionization, though we note that an increased ionization cross-section may increase the rate of surface beam damage in some instances. Precise control over the surfaces of samples is challenging and few atomic-resolution microscopes have the vacuum capabilities standard in surface science, with most instruments operating two to four orders of magnitude above ultra-high vacuum base pressure.

It is important to emphasize that secondary-electron images are obtained simultaneously with other atomic-scale imaging modalities ranging from ADF imaging to chemically specific methods such as electron energy loss spectroscopy and energy dispersive X-ray spectroscopy^1^. Furthermore, unlike proximal scanning probe techniques, there are no issues with inhomogeneous samples, so 3D nanomaterials with unique surface reconstructions^26^ pose no problems. Perhaps most importantly, on the scale of the cost of high-performance electron microscopes, the addition of a secondary-electron detector is minimal, so there are already hundreds of instruments around the world capable of performing analyses similar to what we have described here and further optimization of detector geometries to improve collection efficiency are underway. The good agreement between calculations and experiment demonstrates that HRSEM is a very promising technique. It also opens a door to the study of nanoscale surface electronic states using secondary-electron spectroscopy, which has a wide range of applications including identifying the structural changes of active sites on nanoparticles in catalytic reactions with operando electron microscopy.

## Methods

### Sample preparation

For the electron microscopy work, a 0.7% wt Nb-doped SrTiO_3_ (001) single crystal was purchased commercially (MTI corp) as a 10 × 10 × 0.5 mm wafer. The wafer was cut into standard TEM sample discs with a diameter of 3 mm. The discs were then mechanically thinned to 100 μm using diamond lapping paper and mechanically dimpled at the centre until the centre thickness was ∼30 μm. The samples were then washed in water to remove slurries and soaked in acetone overnight to remove residual mounting waxes. The acetone-cleaned samples were further cleaned by methanol and perforated using a Gatan Precision Ion Polishing System operated at 5 keV with a 10-degree milling angle for 40 min followed by 3 keV milling at 6 degrees for final polishing. Following Ar^+^ ion bombardment, the samples were annealed in air at 1,050 ^o^C for 10 h in a quartz tube furnace. The samples were again baked in air at 300–500 ^o^C for 1–4 h directly before imaging experiments to remove residual contamination.

### Electron microscopy experimental details

HRTEM experiments were performed on the TEAM 0.5 instrument at the NCEM facility of the Molecular Foundry, which is an FEI Titan-class microscope equipped with monochromator and third-order geometric aberration correctors^16^. Focal series of 41 images with a defocus step of −1.05 nm were recorded at an accelerating voltage of 80 kV with an energy spread of 0.1 eV, 0.2 mrad convergence angle, 1.4 nm defocus spread, and the aberration corrector tuned to balance C_3_ against the uncorrected residual C_5_ (C_3_=−16 μm, C_5_=6 mm). Aberration-corrected HRSEM images with simultaneous ADF images were recorded on a Hitachi HD2700C with a cold field-emission source^15^ at the Hitachi Science Laboratory in Hitachi-Naka, Ibaraki, Japan. Data were collected at 200 kV with a convergence angle of 25 mrad and ADF collection semiangles of 53 mrad (inner) and 280 mrad (outer). The SE signal was measured with a Hitachi in-lens Everhart–Thornley SE detector biased at +10 kV. Experiments to determine the energy collection range of the SE detector were performed using a Hitachi single-tilt biasing stage to bias the sample from −20 V to +100 V.

### Processing of experimental HRSEM images

Mean unit cells created over a 60 × 60 nm^2^ field of view were generated by first computing a best-fit linear lattice for each of the four distinct terraces of the ADF micrographs. This lattice was expanded to (6 × 2) unit cells to match the observed symmetry. A mean unit cell for both the ADF and SE micrographs was computed from the (6 × 2) lattice vectors using kernel density estimation (KDE) for a 2D Gaussian kernel with a standard deviation of 1/16th of the unit cell length. To visualize the surface reconstruction in the SE image more easily, a repeated (1 × 1) mean unit cell was extracted from the original data, tiled over the full (6 × 2) unit cell and subtracted in real space from the total (6 × 2) signal to remove all information content with bulk-like periodicities. The resulting processing fully removes the bulk component and is the variation of the surface signal about the same surface signal averaged over the (1 × 1) cell. Finally, all the images were symmetrized assuming a c2 mm plane group. Supplementary Fig. 2 shows the result of this process for four different regions (sample terraces) of this ADF-SE image pair. Note that the same image elements and registration was found for all the regions, indicating that either the top surface is one continuous reconstruction (no steps), or the terrace steps are coherent with the 6 × 2 periodicity and without 90-degree rotation. The application of translational and c2 mm symmetry was critical to the interpretation of the HRSEM experimental data and quantitative comparison with simulations due to a low signal-to-noise compared with more conventional ADF-STEM techniques (see Supplementary Table 6).

### Plan-view HRTEM bulk subtraction

Reduction of the plan-view HRTEM images followed the same procedure as used previously for the silicon 7 × 7 (111) reconstruction^11^. For different defoci, thin areas from the images were extracted and the amplitudes and phases of the two-dimensional reciprocal lattice vectors measured using a cross-correlation method after a Hanning-window FFT. The phase contrast transfer function sin(*χ*) was calculated for each defocus value, and the measured phases of the experimental image were retarded 180 degrees for spatial frequencies for which sin(*χ*)<0. After removal of the bulk reflections, as these would otherwise overwhelm the weaker surface signal, a search was performed over all possible registry shifts for the top and bottom surface structures with the assumption that they were the same. The most consistent registry shift using a conventional R1 metric was then used for further analysis, and inverted assuming a linear-imaging model with the known microscope parameters. Cross-checks were performed using forward multislice simulations using the MacTempas code; a focal spread of 1.4 nm at a thickness of 5.3 nm provided the best match between simulation and experiment. HRTEM image simulations also include a correction for the measured modulation transfer function of the Gatan 894 US1000 CCD detector and a root mean square mechanical vibration of 0.4 Å.

### HRSEM simulation and dielectric screening

We will provide a brief overview of the previous approach, and the extensions to the model we found necessary to include, to properly explain the experimental results.

Many processes can lead to the generation of low-energy (<20 eV) secondary electrons. Some, such as the decay of plasmon excitations are delocalized and so only contribute to the general background, not the atomic details of interest herein. The high-resolution information involves inelastic processes involving localized states. It is well established that for high-energy electron scattering, these processes can be modelled reasonably well via a superposition of the contributions from equivalent neutral atoms; while there are effects due to the change in bonding in the bulk (for example, for SrTiO_3_, see refs 27, 28, 29, 30, 31) and at surfaces^32^,^33^, they are small.

In ref. 4, the triple-differential cross-section involving an incident high-energy plane wave, an inelastically scattered (high-energy) plane wave and a secondary electron ejected from a specific atomistic state was considered. It was pointed out that a scanning probe, as used here, can be considered to be a position-dependent, coherent sum of plane waves. Calculation of the triple-differential cross-section involves transition potentials from the initial bound state of the electron to all possible final states of that electron after ionization (mediated by a Coulomb interaction). Bound states are calculated using a relativistic Hartree–Fock model and unbound final states use a Hartree–Slater potential. The Hartree–Slater potential assumes an atom with N-1 electrons in a relaxed state. While there are some errors in not using the states for the atom in the solid, we verified that (as expected) the differences between the states for the isolated atom and those from DFT calculations of the surface/bulk atoms were not that large. Taking fully relaxed N-1 final states is reasonable as electronic relaxation times are fast. The triple-differential cross-section is then integrated over all possible directions and energies of the fast-scattered electron to yield an angular-dependent cross-section for the ejected (secondary) electrons. This angular dependence is an important part of the physics and cannot be ignored.

It is well known that low-energy electrons undergo multiple elastic scattering events while exiting the sample surface, which cannot be ignored in the case of angle-resolved techniques such as LEED and ARPES^34^,^35^,^36^,^37^. The attenuation of escaping secondary electrons is also modulated by the angular variance of the semi-infinite band structure^38^,^39^. However, angular and energy integration performed by the in-lens secondary-electron detector used here greatly ameliorates signal variance due to the diffraction and electronic structure of secondary electrons as they exit the sample. HRSEM is thus a primarily incoherent imaging technique. It is also worth noting that even at the low energies of secondary emission (<50 eV), elastic and inelastic scattering processes associated with the secondary electron are heavily weighted in the forward direction^40^,^41^,^42^. Therefore it is appropriate in this context to treat the attenuation of the ejected electrons as a function of the path length to the surface, dependent on the angle of ejection and weighted by the cross-section for emission of the secondary electron in that particular direction. The inelastic mean free path of the ejected electrons is given, as a function of energy, by equation (13) in ref. 43, with a monolayer thickness a=1.2 nm.

Previously, only core shells were incorporated into the model. Here, we incorporate semicore and valence electrons where the screening associated with other nearby atoms and virtual plasmon excitations become important.

The problem of the potential seen by a charge near a surface has been extensively studied both because of an interest to fundamental physics and because it matters for many surface spectroscopies. We borrow here from standard results for the solution of Poisson's equation for aloof scattering often performed in transmission electron microscopes^25^,^44^,^45^ and the non-relativistic case for low-energy electron energy loss as recently reviewed by Hogan *et al.*^46^ We consider the simple case of a half-plane where for *z*>0, we have a bulk dielectric, and for *z*<0, vacuum. Quoting from equations (37) and (38) of the work by Hogan *et al.*^46^, for a bulk material with a dielectric constant of *ɛ*, both the classical electrostatic method of image charges and the dielectric formalism give the same result for the potential 
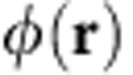
 at some position 
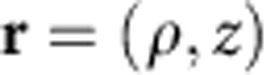
 due to a point charge at (0,0,z_0_) inside a continuum crystal:


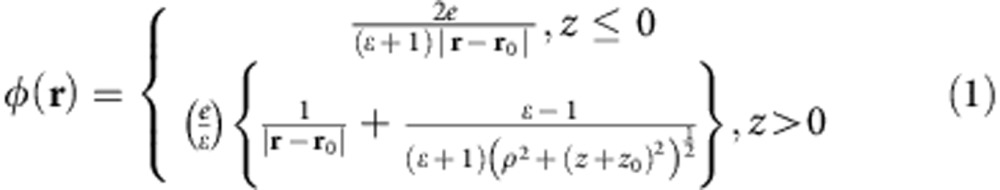


Deep inside the crystal (*z*>>0), the second term becomes negligible and the first term reduces to that for conventional screening of:


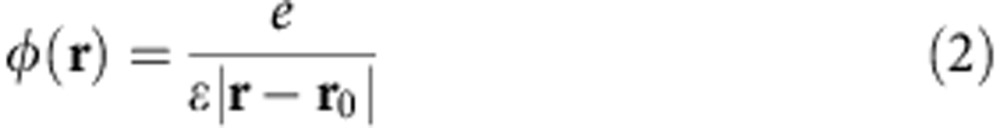


As we approach the surface, both terms reduce to the form for **r**_**0**_=0:


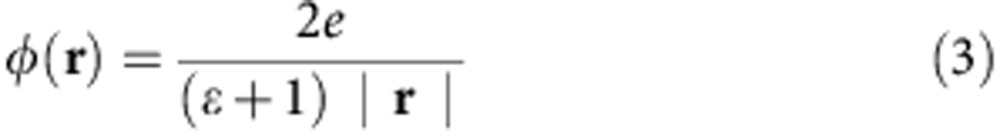


For inelastic scattering, one uses the dissipative part of the potential, the imaginary part within the context of an optical potential. Here, we are concerned with the real part used to convert the change in charge density due to a secondary-electron excitation into a potential that couples with the incident electron beam when the three-particle cross-section is calculated. We argue that it is then more correct to use the appropriate frequency-dependent dielectric constant *ɛ*(Δ*E*/*ħ*,**q**) for the total energy loss Δ*E* with **q** the wave number dependence, that is, we include implicitly the time dependence. The **q** dependence will lead to a conventional exponential screening of long-range contributions, which explicit calculations indicated played a lesser role.

Hence, in the limit of excitations only in the bulk, the screening of the primary inelastic scattering event will be 
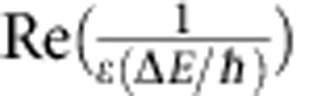
 and in the limit of only surface screening it will be 
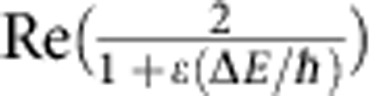
. For completeness, more complicated forms as discussed by Hogan *et al.*^46^ could be used; we will leave this as a topic for future research particularly when energy-resolved imaging of the secondary electrons becomes possible.

Using the energy-dependent complex dielectric function reported in ref. 25, which was obtained through a Kramers–Kronig transformation of experimental electron energy loss spectra of SrTiO_3_, the bulk and surface limits were calculated and normalized to unity for energies >60 eV. The results for the screening are plotted in Supplementary Fig. 7. The energy transfer term was the summation of the orbital band edge energies relative to the valence band edge as calculated from DFT, the DFT work function of 8.16 eV (see Supplementary Discussion) and a secondary-electron escape energy of 10 eV. The resultant damping coefficients are given in Supplementary Table 2 for the core and semicore states of SrTiO_3_. The average values were used in the simulations. The main consequence of the screening is that for relatively low escape energies, the states near the Fermi energy such as the oxygen 2*p* states are strongly but not completely damped, whereas there is minimal damping of the semicore or core states. Supplementary Fig. 6 compares an HRSEM simulation of the Sr7 structure to experiment without accounting for the proper dielectric damping of the inelastic process. The simulation is dominated by the oxygen signal, and is unable to reproduce the experimental result. Compare Supplementary Fig. 6 to Fig. 4 in the main text, which fully accounts for the dielectric damping and provides a much better match to the experimental data.

Because of the inverse energy dependence of the inelastic scattering cross-section, very deep core states (for example, >2 keV) do not contribute significantly to the images. However, the higher energy core states such as the O 1*s* as well as semicore states such as the O 2*s* can, as well as valence states such as O 2*p*. In earlier work^4^, it was argued that the valence states were delocalized and so did not contribute. While that assumption was valid for a bulk YBa_2_Cu_3_O_7−*x*_ crystal with unknown surface termination (presumably amorphous from sample preparation), it is not an adequate description here for the case of a 0.39 nm thick well-ordered surface reconstruction. Note that the dielectric screening for a bulk signal is larger than that at a surface, and amorphous material and carbonaceous contaminants on the bulk YBa_2_Cu_3_O_7−*x*_ crystal will introduce more screening, further diminishing valence contributions. In the present case, simulations completely excluding the valence states were not in agreement with the bulk-subtracted signal (see Fig. 4). A Pearson product–moment correlation score,


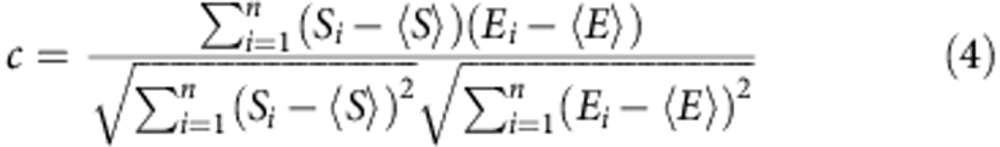


where *S* and *E* are the simulated and experimental image pixel values, respectively, was computed comparing the bulk-subtracted HRSEM simulations to experimental data for the inclusion of only the core states as done in earlier work^4^, adding semicore orbitals, and adding valence orbitals. Correlation plots are shown in Fig. 4 and the correlation scores are displayed in Supplementary Table 4. We have also calculated the relative contributions to the total simulated HRSEM image from each orbital as shown in Supplementary Table 3. Over 90% of the total simulated HRSEM signal arises from the semicore and valence orbitals.

For completeness, we note that there are some approximations in this approach, and, in principle, a more complete time-dependent DFT or similar model could be used taking into proper account the surface geometry. There is also a large amount of interesting science that stems from this analysis, particularly if one considers detectors capable of resolving the energies of the escaping secondaries; we leave this to future work.

Finally, the HRSEM simulations, using the experimental parameters, took into account dynamical scattering of the incident probe. Finite source size and loss of spatial resolution, due to terrace averaging and so on, was taken into account by convolving with a symmetric 2D Gaussian with a full-width at half-maximum of 0.14 nm.

### Surface X-ray diffraction refinement

The top three layers (24 unique sites) were refined using the Shelx program against the previously published surface X-ray diffraction data^19^. The metal positions were very stable, the oxygen positions less so and, to avoid artifacts, an anti-bumping constraint that the metal-oxygen distances had to be >0.17 nm was used. The top four Ti and the Sr atoms were refined using anisotropic temperature factors, otherwise isotropic ones were used with the oxygen atoms in the bottom layer constrained to have the same temperature factor. While the temperature factors for the metal atoms not at special sites are reasonable, those at special sites are anomalously large, which is an indicator of disorder.

To generate the effective 2D positions, only the (hk0) reflections were used and the outermost four Ti and the Sr atom refined using the EDM code with isotropic temperature factors, a comparison being given in Supplementary Table 5.

### DFT calculations

DFT calculations were performed with the all-electron augmented plane wave + local orbitals WIEN2K code^47^. The surface in-plane lattice parameters were set to those for the corresponding DFT-optimized bulk cell, with ∼1.6 nm of vacuum to avoid errors within the DFT calculations as well as in the STM simulations, the later being done using the Tersoff–Hamann approximation^48^. Muffin-tin radii were set to 1.55, 2.36 and 1.75 Bohrs for O, Sr and Ti, respectively, as well as a min (RMT)*K_max_ of 7.0, with a 3 × 3 × 1 Brillouin-zone reciprocal space sampling of the primitive unit cell. The electron density and atomic positions were simultaneously converged using a quasi-Newton algorithm^49^; the numerical convergence was better than 0.01 eV (1 × 1 cell)^−1^ surface cell. The PBEsol^50^ generalized gradient approximation as well as the revTPSS method^51^ was used with 0.5 on-site exact exchange, the optimized number for several test TiO_*x*_ molecules similar to earlier work^22^. The surface enthalpy for each (1 × 1) surface unit cell (*E*_surf_) was calculated as: *E*_surf_=(*E*_slab_−*E*_STO_*N*_STO_−*E*_TO_*N*_TO_)/(2*N*_1 × 1_), where *E*_slab_ is the total enthalpy of the slab, *E*_STO_ for one bulk SrTiO_3_ unit cell, *N*_STO_ the number of bulk SrTiO_3_ unit cells, *E*_TO_ bulk rutile TiO_2_, *N*_TO_ the number of excess TiO_2_ units and (*N*_1 × 1_) the number of (1 × 1) cells. Consistency checks between the different functionals indicated an error in the energies of ∼0.1 eV (1 × 1 cell)^−1^ (∼60 mJ m^−2^, 8 kJ mol^−1^). STM simulations were performed averaged over the lower 1 eV of unoccupied states with a Gaussian smearing of 0.3 au in the *xy* image plane and 0.05 au vertical to model tip vibration, using code written by LDM that is part of the latest release of the Wien2k software.

## 

## Additional information

**How to cite this article:** Ciston, J. *et al.* Surface determination through atomically resolved secondary-electron imaging. *Nat. Commun.* 6:7358 doi: 10.1038/ncomms8358 (2015).

## Supplementary Material

Supplementary Information Supplementary Figures 1-7, Supplementary Tables 1-6, Supplementary Note 1, Supplementary Discussion and Supplementary References
