# Supplementary material for: Surface determination through atomically resolved secondary-electron imaging
Source: Nat Commun. 2015 Jun 17;6:7358. doi: 10.1038/ncomms8358 (PMC4557350; doi:10.1038/ncomms8358)
Supplement: Supplementary Information — Supplementary Figures 1-7, Supplementary Tables 1-6, Supplementary Note 1, Supplementary Discussion and Supplementary References [file ncomms8358-s1.pdf]

Supplementary Figures:

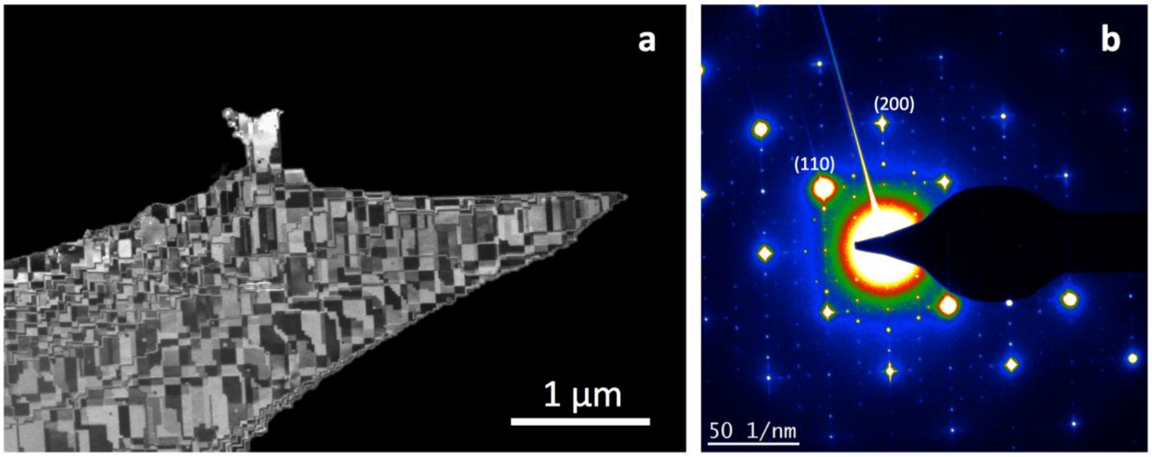

Supplementary Fig. 1: a. Weak beam dark field image of SrTiO3 001 c(6×2) single crystal with g=(200) used for imaging and 3g=(600) strongly excited, **b.** Transmission electron diffraction pattern of the c(6×2) reconstruction acquired approximately 3 degrees off-zone and away from both 2-beam and weak beam conditions to minimize bulk dynamical diffraction. Strong bulk reflections are labeled, while the weaker reflections of higher periodicity are due to the surface reconstruction.

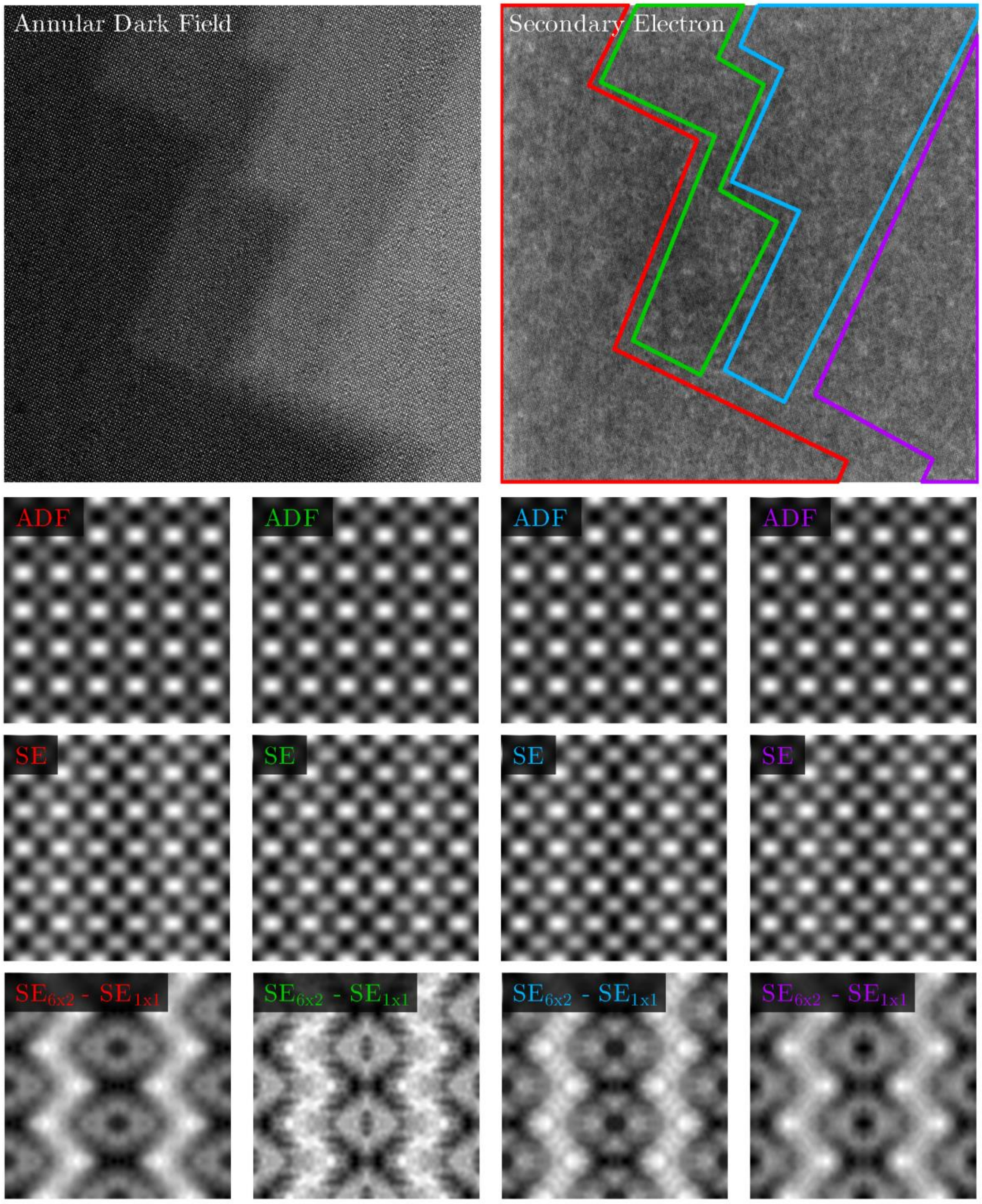

Supplementary Fig. 2. (top) Unprocessed ADF and SE micrographs, with 4 sub-region terraces overlaid on SE image with a 60 × 60 nm^2 field of view. (bottom) Mean (6×2), ADF, SE and SE with (1×1) cell subtracted unit cells corresponding to the 4 colored sub-regions with a 2.34 × 2.34 nm^2 field of view.

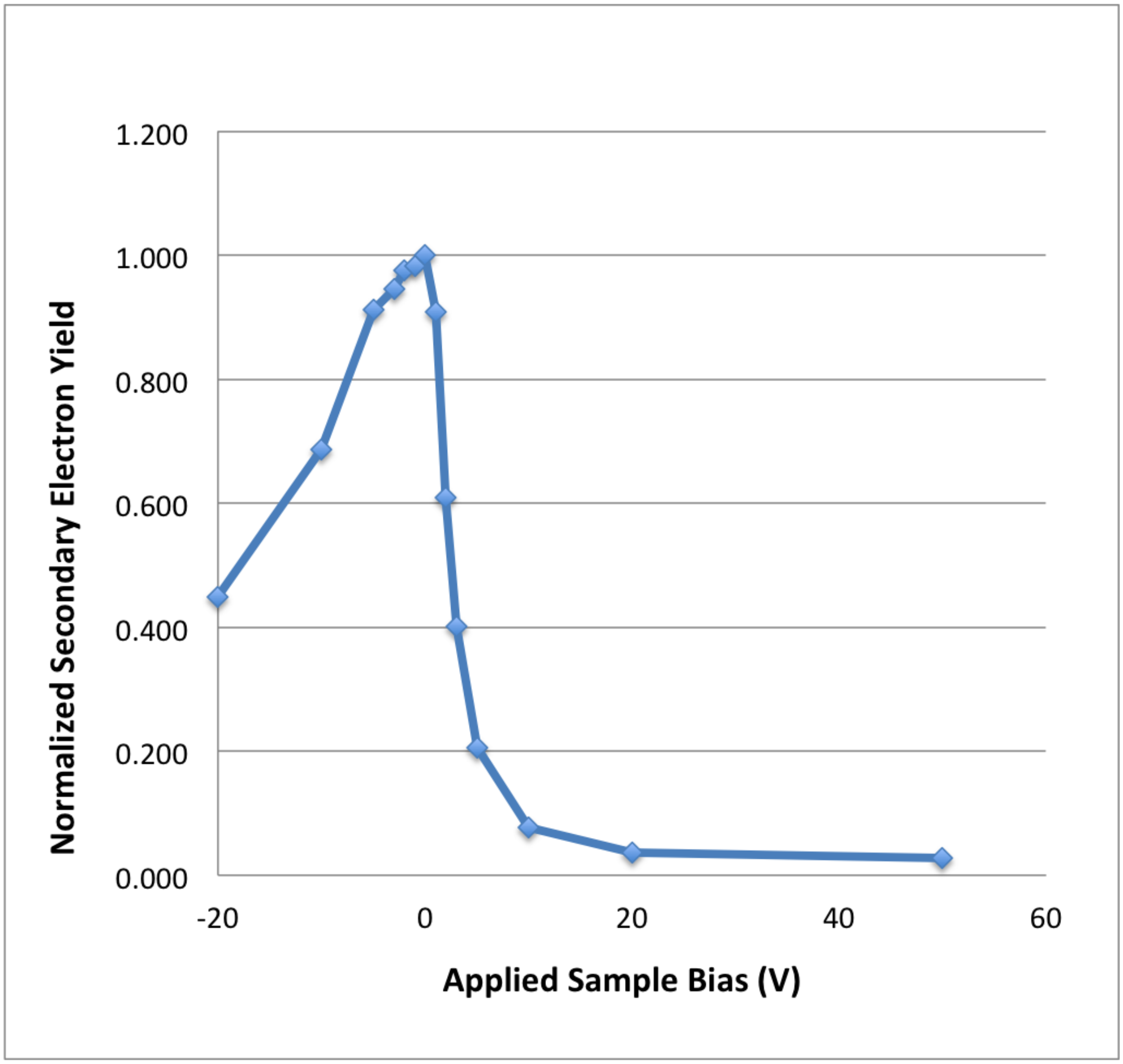

Supplementary Fig. 3. Experimental normalized secondary electron detector yield as a function of sample bias for the Hitachi in-lens SE detector geometry.

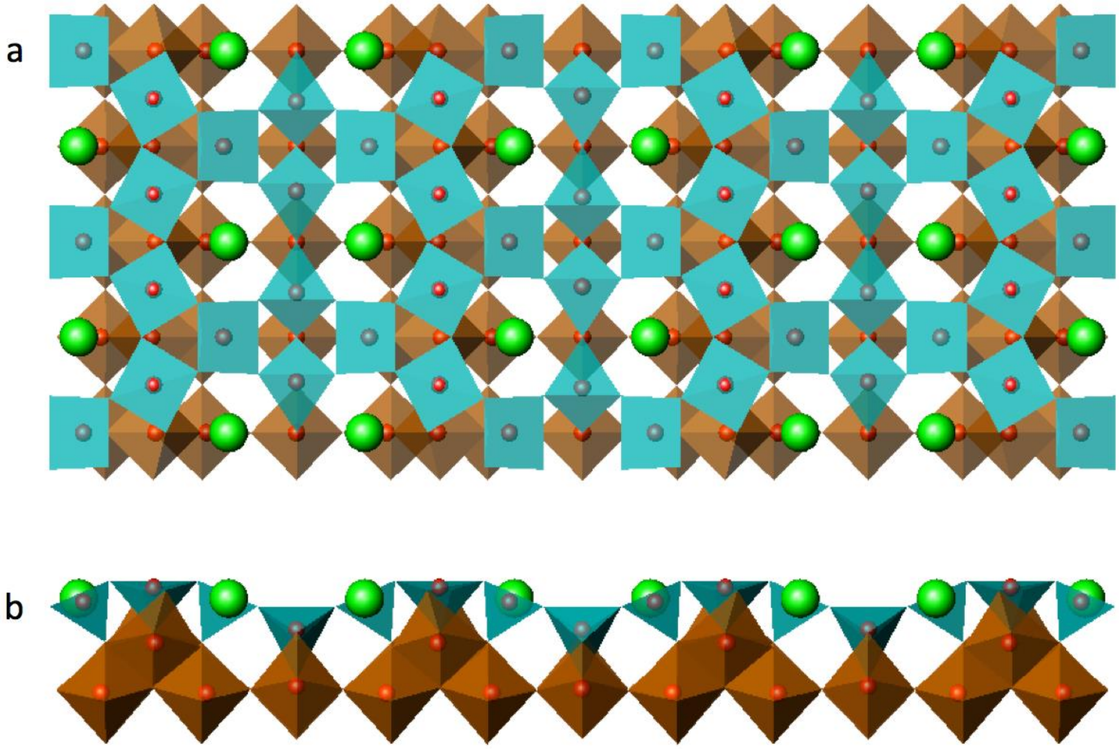

Supplementary Fig. 4. Polyhedral representation of the $SrTiO_3$-(100)-c(6×2) Sr7 reconstruction; brown TiO_6 octahedra, blue TiO_5 units with the Ti atoms in red and the green spheres are Sr atoms. **a.** viewed from above the surface, the direction of the HRSEM and HRTEM images **b.** a side view.

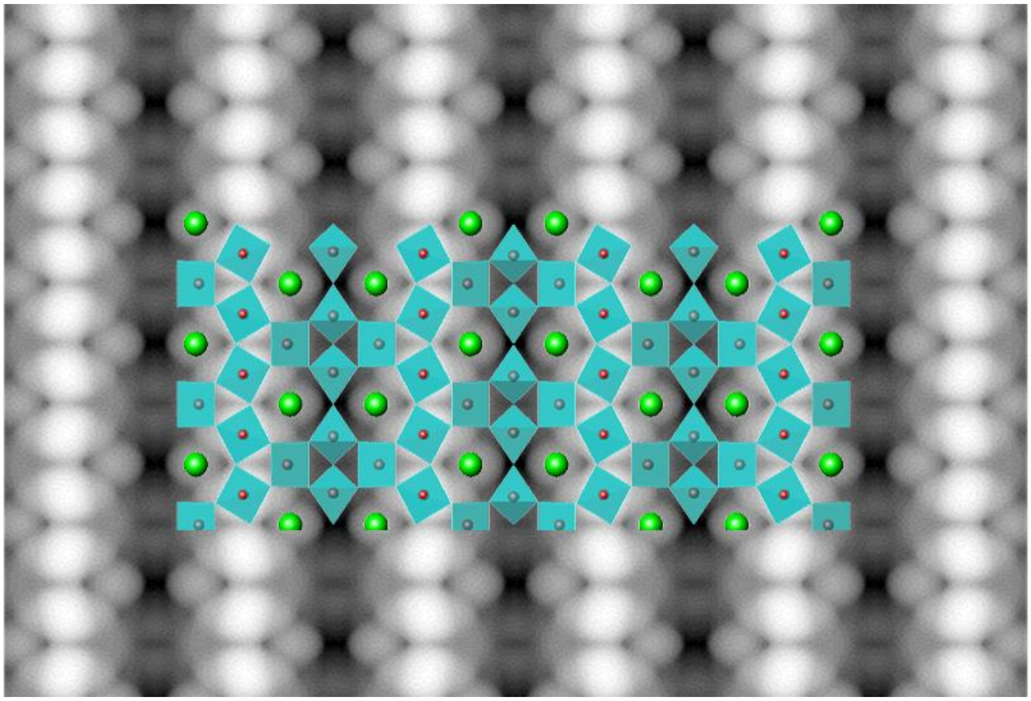

Supplementary Fig. 5. Empty state STM simulation with a polyhedral model of the outermost layer superimposed. The cyan units are TiO_5 polyhedra, large green spheres are Sr atoms. This image is consistent with the experimental images published to date, although they are lower resolution and show disorder. The similarity to the HRSEM image is a partial co-incidence; the atoms which have intermediate energy semi-core states are also those where the empty state density is higher; in general there will not necessarily be many similarities.

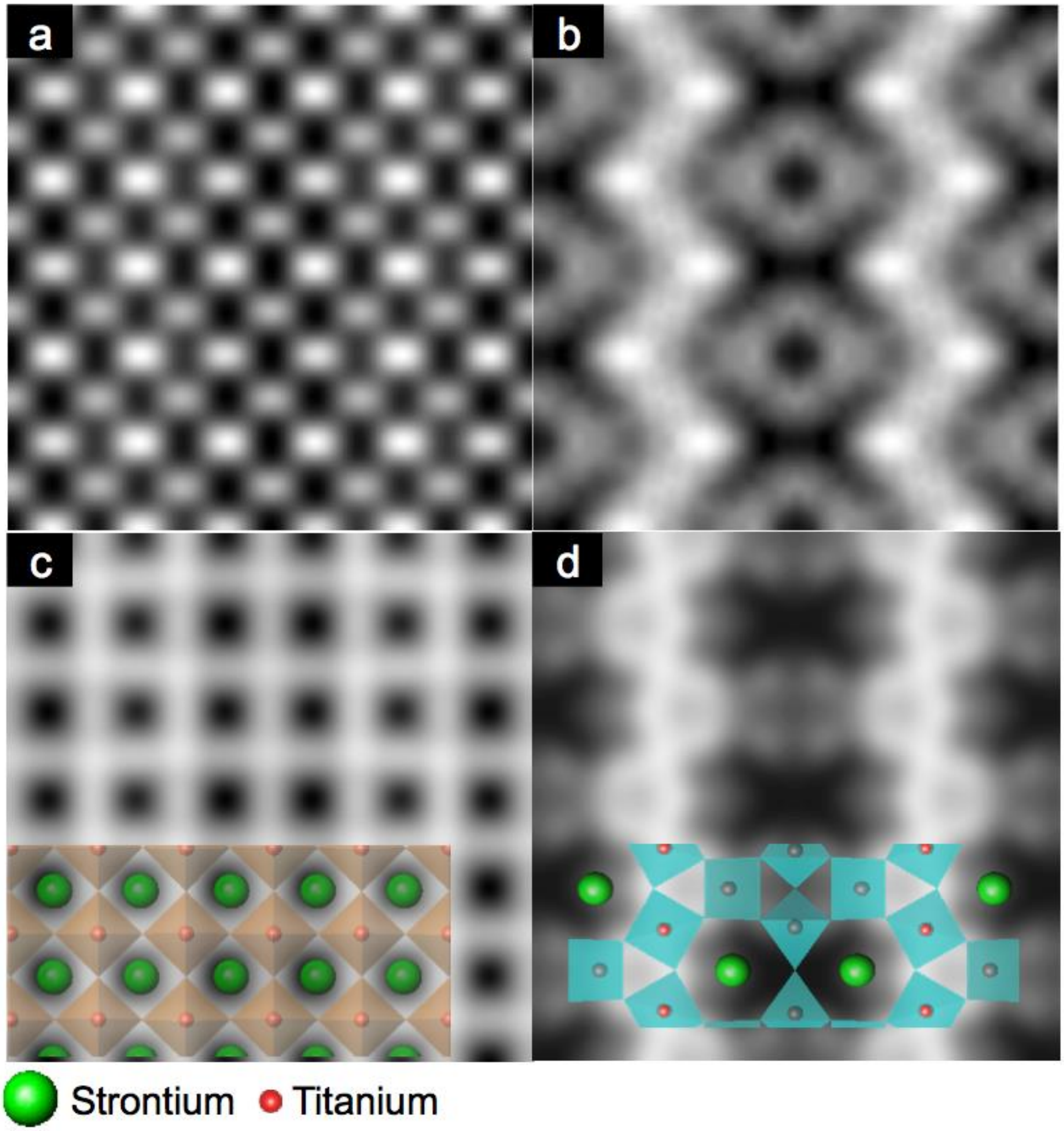

Supplementary Fig. 6. HRSEM experiment and simulation without dielectric screening terms: **a.** Experiment – total image with translational 6×2 unit cell averaging and cmm symmetry applied **b.** Experiment – bulk subtracted **c.** Simulation – total image including contributions from all orbitals, but without the correction for local dielectric screening (with surface and bulk structure overlayed) **d.** Simulation – bulk subtracted.

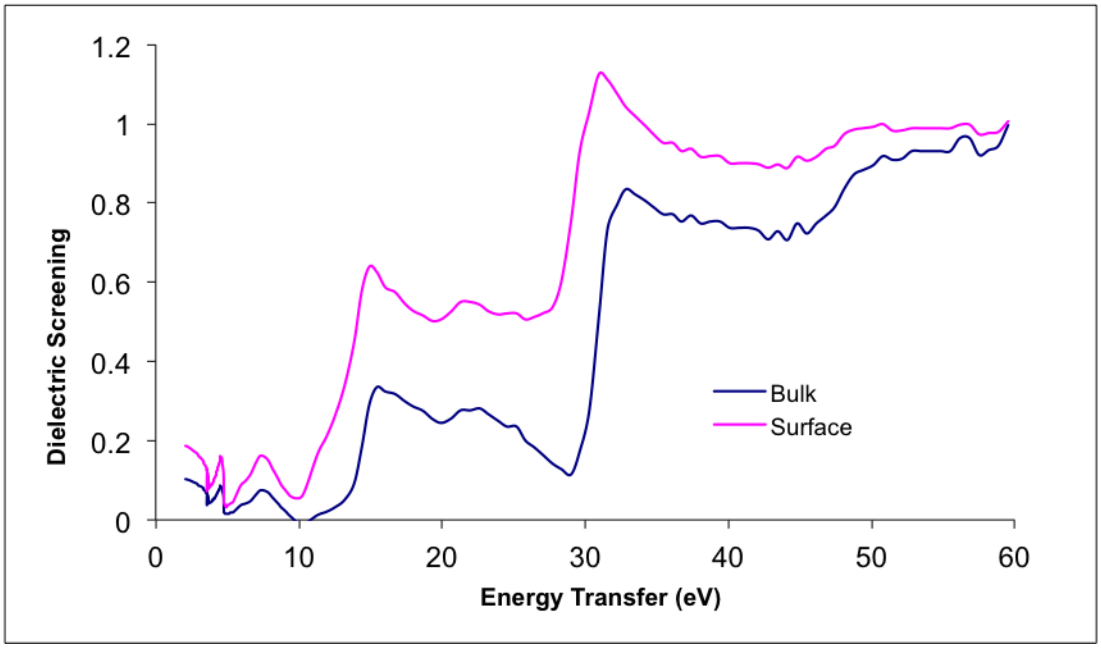

Supplementary Fig. 7. Energy-dependent dielectric screening coefficients for bulk and surface limits of the dielectric screening in $SrTiO_3$

Supplementary Tables:

		Sr7: DFT Positions				Sr7: SXRD Refined Positions			
		X	Y	Z		X	Y	Z	Uiso
O0001	O	0.32028	0.17239	0.34599		0.35316(12)	0.18416(2)	0.33577(10)	0.028(12)
Ti002	Ti	0.25000	0.25000	0.34027		0.25000	0.25000	0.339574(8)	0.41(3)
O0003	O	0.00000	0.22911	0.33818		0.00000	0.226(3)	0.3596(9)	0.000(9)
Sr004	Sr	0.00000	0.11932	0.33105		0.00000	0.12077(11)	0.3309(2)	0.0316(8)
Ti005	Ti	0.00000	0.37292	0.32806		0.00000	0.39014(2)	0.32853(6)	0.006(3)
O0006	O	0.19394	0.43196	0.32421		0.18093(6)	0.43317(3)	0.32391(7)	0.000(8)
Ti007	Ti	0.26568	0.00000	0.30468		0.250(4)	0.00000	0.3068(4)	0.045(10)
O0008	O	0.00000	0.00000	0.30244		0.00000	0.00000	0.3053(16)	0.000(16)
O0009	O	0.25000	0.25000	0.30168		0.25000	0.25000	0.303117(8)	0.9(5)
O0010	O	0.00000	0.50000	0.29467		0.00000	0.50000	0.305(6)	0.11(8)
O0011	O	0.00000	0.33433	0.29393		0.00000	0.32609(2)	0.29417(11)	0.25(17)
Ti012	Ti	0.00000	0.24709	0.29334		0.00000	0.25187(2)	0.29743(13)	0.18(2)
O0013	O	0.00000	0.16747	0.28988		0.00000	0.1544(2)	0.287956(9)	0.07(3)
O0014	O	0.24878	0.00000	0.26263		0.266(10)	0.00000	0.2546(12)	0.016(8)
Ti015	Ti	0.00000	0.00000	0.25729		0.00000	0.00000	0.252(2)	0.11(3)
O0016	O	0.00000	0.25268	0.25571		0.00000	0.256955(19)	0.2521(4)	0.016(8)
O0017	O	0.00000	0.08007	0.25477		0.00000	0.086(3)	0.2608(17)	0.016(8)
Ti018	Ti	0.00000	0.50000	0.25456		0.00000	0.50000	0.2510(3)	0.029(10)
O0019	O	0.00000	0.41887	0.25313		0.00000	0.42535(2)	0.2498(3)	0.016(8)
Ti020	Ti	0.00000	0.34198	0.25138		0.00000	0.33162(2)	0.2531(4)	0.07(2)
Ti021	Ti	0.00000	0.15695	0.25077		0.00000	0.1606(3)	0.251627(9)	0.10(2)
O0022	O	0.25003	0.33555	0.24466		0.226(7)	0.3288(16)	0.2444(12)	0.016(8)

Supplementary Table 1. Comparison of Sr7 atomic coordinates relaxed by DFT and refined against 3D surface x-ray diffraction data. Surface unit cell parameters: a = 7.81 Å, b = 23.43 Å, c = 48.00 Å.

Orbital	ΔE (eV)	Bulk	Surface	Average	# Electrons
O2p	20.66	0.25	0.52	0.39	6
O2s	35.16	0.77	0.95	0.86	2
Sr 4p	33.16	0.83	1	0.92	6
Sr 4s	51.16	0.91	0.98	0.95	2
Ti 3p	54.16	0.93	0.99	0.96	6

Supplementary Table 2. Calculated energy-dependent dielectric damping coefficients for semicore and valence states in $SrTO_3$. The average values have been used in the HRSEM calculations. All lower-lying (more tightly bound) orbitals are assumed to have damping coefficients of unity

	Ti	Sr	O
1s	2.60E-05	1.15E-06	0.0058
2s	0.0013	6.12E-05	**0.1391**
2p	0.0138	5.83E-04	**0.4406**
3s	**0.0174**	0.0010	-
3p	**0.1268**	0.0089	-
3d	-	0.0643	-
4s	-	**0.0165**	-
4p	-	**0.1638**	-

Supplementary Table 3. Fractional contributions from each of the total simulated HRSEM signal for the SrTiO3 (100) c(6×2) structure as represented in Figure 4c. Values in bold represent valence and semi-core orbitals and account for 90% of the total simulated HRSEM signal.

Sr7 (core)	Sr7 (semicore)	Sr7 (full)	Sr7-effective (full)	Ref 1 (full)
0.19	0.62	0.75	0.95	0.57

Supplementary Table 4. Pearson product-moment correlation values comparing the experimental bulk-subtracted data to simulated HRSEM values for the present structure simulated using only the core states (up to and including the 3d state in Sr, the 2p in Ti and 1s in oxygen) semicore states (adding the 4s and 4p for Sr, the 3s and 3p for Ti and 2s for O) and full valence states (adding the contribution for a filled 2p orbital in O). Correlation score for the full HRSEM simulation of the Sr7-effective structure and the Rumpled Vacancy structure[1] is shown for comparison.

	DFT		3D SXRD		2D SXRD	
	x	y	x	y	x	y
Ti002	0.25	0.25	0.25	0.25	0.25	0.25
Sr004	0	0.11932	0	0.12077(11)	0	0.15041
Ti005	0	0.37292	0	0.39014(2)	0	0.40809
Ti007	0.26568	0	0.250(4)	0	0.17608	0
Ti012	0	0.24709	0	0.25187(2)	0	0.2504

Supplementary Table 5. Comparison of outer layer metal atoms from the DFT, 3D-SXRD and 2D SXRD refinements. Surface unit cell parameters: a = 7.81 Å, b = 23.43 Å

	Raw Image	Mean 6×2 unit cell	cmm Symmeterization
**ADF-STEM**	1.22	28.9	32.5
**HRSEM**	0.26	4.2	7

Supplementary Table 6. Root mean square Signal/Noise ratios measured from ADF-STEM and HRSEM experimental images before and after application of translational symmetry (6×2 mean unit cell) and enforcement of c2mm symmetry (determined from x-ray and electron diffraction experiments).

Supplementary Note 1: $SrTiO_3$ (001) c(6×2) Sr7 structure in Crystallographic Information File (CIF) format

```
data_Wien2k_Data
_cell_length_a    7.820013
_cell_length_b   23.458653
_cell_length_c   47.999962
_cell_angle_alpha   90.000000
_cell_angle_beta    90.000000
_cell_angle_gamma   90.000000
_cell_measurement_temperature 0.0
_diffrn_ambient_temperature 0.0
_symmetry_space_group_name_H-M         'Cmmm   '
_symmetry_space_group_number   65
_refine_date  ' 8- 2-2015'
_refine_method 'generated from Wien2k code'
_refine_special_details
;
Structure converted from Wien2k struct file, Version 9.1
;

loop_
_symmetry_equiv_pos_as_xyz
   +x,+y,+z
   -x,-y,-z
   -x,-y,+z
   -x,+y,-z
   -x,+y,+z
   +x,-y,-z
   +x,-y,+z
   +x,+y,-z
   +x+1/2,+y+1/2,+z
   -x+1/2,-y+1/2,-z
   -x+1/2,-y+1/2,+z
   -x+1/2,+y+1/2,-z
```

```
  -x+1/2,+y+1/2,+z
  +x+1/2,-y+1/2,-z
  +x+1/2,-y+1/2,+z
  +x+1/2,+y+1/2,-z
loop_
_atom_site_label
_atom_site_type_symbol
_atom_site_fract_x
_atom_site_fract_y
_atom_site_fract_z
_atom_site_U_iso_or_equiv

O0001 O   0.320277   0.172386   0.345992   0.05
Ti002 Ti  0.250000   0.250000   0.340271   0.05
O0003 O   0.000000   0.229108   0.338183   0.05
Sr004 Sr  0.000000   0.119325   0.331050   0.05
Ti005 Ti  0.000000   0.372921   0.328063   0.05
O0006 O   0.306058   0.068038   0.324210   0.05
Ti007 Ti  0.265676   0.000000   0.304684   0.05
O0008 O   0.000000   0.000000   0.302437   0.05
O0009 O   0.250000   0.250000   0.301684   0.05
O0010 O   0.000000   0.500000   0.294673   0.05
O0011 O   0.000000   0.334327   0.293933   0.05
Ti012 Ti  0.000000   0.247087   0.293338   0.05
O0013 O   0.000000   0.167469   0.289878   0.05
O0014 O   0.248776   0.000000   0.262629   0.05
Ti015 Ti  0.000000   0.000000   0.257293   0.05
O0016 O   0.000000   0.252681   0.255713   0.05
O0017 O   0.000000   0.080065   0.254774   0.05
Ti018 Ti  0.000000   0.500000   0.254561   0.05
O0019 O   0.000000   0.418873   0.253134   0.05
Ti020 Ti  0.000000   0.341985   0.251380   0.05
Ti021 Ti  0.000000   0.156945   0.250771   0.05
O0022 O   0.250033   0.335546   0.244665   0.05
O0023 O   0.000000   0.000000   0.214155   0.05
O0024 O   0.000000   0.500000   0.211751   0.05
O0025 O   0.000000   0.334174   0.208189   0.05
O0026 O   0.000000   0.166035   0.207343   0.05
Ti027 Ti  0.250567   0.167347   0.204251   0.05
Ti028 Ti  0.246191   0.000000   0.202975   0.05
O0029 O   0.250911   0.418210   0.200444   0.05
O0030 O   0.250000   0.250000   0.199036   0.05
Sr031 Sr  0.000000   0.414501   0.164204   0.05
Sr032 Sr  0.000000   0.250016   0.164118   0.05
O0033 O   0.238092   0.000000   0.163915   0.05
Sr034 Sr  0.000000   0.086052   0.163757   0.05
```

O0035 O 0.245475 0.334979 0.163232 0.05
O0036 O 0.000000 0.500000 0.124962 0.05
O0037 O 0.000000 0.166578 0.123450 0.05
Ti038 Ti 0.249825 0.333307 0.122530 0.05
Ti039 Ti 0.250008 0.000000 0.122280 0.05
O0040 O 0.000000 0.333429 0.122017 0.05
O0041 O 0.250000 0.250000 0.122331 0.05
O0042 O 0.000000 0.000000 0.121450 0.05
O0043 O 0.249991 0.416939 0.121979 0.05
Sr044 Sr 0.000000 0.249968 0.081762 0.05
O0045 O 0.259995 0.000000 0.081743 0.05
Sr046 Sr 0.000000 0.416372 0.081724 0.05
Sr047 Sr 0.000000 0.083640 0.081727 0.05
O0048 O 0.254338 0.333231 0.081638 0.05
O0049 O 0.000000 0.333347 0.041525 0.05
O0050 O 0.000000 0.000000 0.042457 0.05
O0051 O 0.000000 0.166647 0.040122 0.05
O0052 O 0.000000 0.500000 0.039349 0.05
O0053 O 0.250009 0.416712 0.040811 0.05
O0054 O 0.250000 0.250000 0.040691 0.05
Ti055 Ti 0.249991 0.333314 0.040824 0.05
Ti056 Ti 0.250012 0.000000 0.040798 0.05
Sr057 Sr 0.000000 0.249999 0.000000 0.05
O0058 O 0.240518 0.000000 0.000000 0.05
Sr059 Sr 0.000000 0.416483 0.000000 0.05
Sr060 Sr 0.000000 0.083562 0.000000 0.05
O0061 O 0.245676 0.333502 0.000000 0.05
#End data_Wien2k_Data

Supplementary Discussion

Convex Hull Construction: The convex hull is the multidimensional surface connecting the lowest energy structures as a function of composition and thermodynamic state variables compared to some reference states, here the enthalpy as a function of composition in units of excess TiO_2 units in every 1×1 surface unit cell referenced to bulk TiO_2 (rutile) and bulk $SrTiO_3$. Any structure which lies above the convex hull should thermodynamically decompose into a one or a mixture of structures on the convex hull. For instance, ignoring the error bars in Figure 3 then the TiO_2 1×1 structure should decompose into a mixture of the SrO 1×1 and √13×√13 structures (or SrO 1×1 + 3×3), which is consistent with experimental results[2]. A significant advantage of the convex-hull approach is that systematic errors in the energies of reference only lead to a linear shift of

the energies and therefore do not effect what structures lie on the convex hull, i.e. the predicted thermodynamically stable structures.

We use error bars as DFT has limits particularly with what are called strongly-correlated oxides. Common, simple functionals badly overestimate the covalency, leading to too much hybridization of the oxygen 2p and metal d states. While it is common to use LDA+U methods to correct this, we prefer an on-site exact exchange method as this leads to an effective U which varies as a function of metal co-ordination so is more appropriate. In addition to this, the use of a metaGGA leads to a much better treatment of the states at surfaces, and much better surfaces errors. However, there will still be systematic errors, for instance the non-bonded O-O repulsions are probably under-estimated. For these reasons we use an error bar of 0.1 $eV(1\times1\ cell)^{-1}$ surface unit cell which is reasonable, perhaps slightly conservative.

DFT Work functions: Work functions are sensitive to the exact structure of a surface. To our knowledge the work function for the c(6×2) reconstruction has not been measured, only a value of 4.2 eV for a reduced (100) surface of $SrTiO_3$[3] referenced to a Fermi level of 0.2 eV above the conduction band edge. Correcting for an experimental indirect band gap of 3.25 eV[4] and the Fermi level offset gives a value of 7.65 eV for a reduced surface relative to the valence band edge. The DFT work function of 8.16 eV for the c(6×2) reconstruction is in reasonable agreement particularly as this is for a stable reconstruction where there is more bonding at the surface compared to reduced bulk-terminated $SrTiO_3$ and hence one expects a larger work function. For completeness, what matters herein is a work function referenced to the valence band edge rather than to the Fermi energy since this is the value needed when calculating the total energy loss associated with a secondary electron excitation. Conventional DFT is good for ground-state levels, not so good in many cases for excited states. The band gap is intrinsically an excited-state property, and unless one uses methods such as hybrids with exact exchange or GW which includes unoccupied state terms, the band gap is not correctly modeled. In contrast, the work function corresponds to the difference in energy between the highest occupied ground state and vacuum, with the vacuum energy implicitly included in a surface slab calculation. The work function is therefore in general quite well modeled by DFT, although it is not perfect.

Supplementary References:

1 Lanier, C. H. *et al.* Atomic-scale structure of the $SrTiO_3$(001)-c(6×2) reconstruction: Experiments and first-principles calculations. *Phys Rev B* **76**, 045421 (2007).

2 Lin, Y. Y. *et al.* Synthesis-dependent atomic surface structures of oxide nanoparticles. *Phys Rev Lett* **111**, 156101 (2013).

3 Chung, Y. W. & Weissbard, W. B. Surface spectroscopy studies of the $SrTiO_3$ (100) surface and the platinum-$SrTiO_3$ (100) interface. *Phys Rev B* **20**, 3456-3461 (1979).

4 van Benthem, K., Elsässer, C. & French, R. H. Bulk electronic structure of SrTiO3: Experiment and theory. *J. Appl. Phys.* **90**, 6156-6164 (2001).
